# Generalized method of moments for estimating parameters of stochastic reaction networks

**DOI:** 10.1186/s12918-016-0342-8

**Published:** 2016-10-21

**Authors:** Alexander Lück, Verena Wolf

**Affiliations:** Department of Computer Science, Saarland University, Campus E 13, Saarbrücken, 66123 Germany

**Keywords:** Biochemical reaction network, Stochastic model, Parameter estimation, Generalized method of moments

## Abstract

**Background:**

Discrete-state stochastic models have become a well-established approach to describe biochemical reaction networks that are influenced by the inherent randomness of cellular events. In the last years several methods for accurately approximating the statistical moments of such models have become very popular since they allow an efficient analysis of complex networks.

**Results:**

We propose a generalized method of moments approach for inferring the parameters of reaction networks based on a sophisticated matching of the statistical moments of the corresponding stochastic model and the sample moments of population snapshot data. The proposed parameter estimation method exploits recently developed moment-based approximations and provides estimators with desirable statistical properties when a large number of samples is available. We demonstrate the usefulness and efficiency of the inference method on two case studies.

**Conclusions:**

The generalized method of moments provides accurate and fast estimations of unknown parameters of reaction networks. The accuracy increases when also moments of order higher than two are considered. In addition, the variance of the estimator decreases, when more samples are given or when higher order moments are included.

**Electronic supplementary material:**

The online version of this article (doi:10.1186/s12918-016-0342-8) contains supplementary material, which is available to authorized users.

## Background

A widely-used approach in systems biology research is to design quantitative models of biological processes and refine them based on both computer simulations and wet-lab experiments. While a large amount of sophisticated parameter inference methods have been proposed for deterministic models, only few approaches allow the efficient calibration of parameters for large discrete-state stochastic models that describe stochastic interactions between molecules within a single cell. Since research progress in experimental measurement techniques that deliver single-cell and single-molecule data has advanced, the ability to calibrate such models is of key importance. For instance, the widely-used flow cytometric analysis delivers data from thousands of cells which yields sample means and sample variances of molecular populations.

Here, we focus on the most common scenario: a discrete stochastic model of a cellular reaction network with unknown reaction rate constants and population snapshot data such as sample moments of a large number of observed samples. The state of the model corresponds to the vector of current molecular counts, i.e., the number of molecules of each chemical species, and chemical reactions trigger state transitions by changing the molecular populations. A system of ordinary differential equations, the chemical master equation [1], describes the evolution of the state probabilities over time.

A classical maximum likelihood (ML) approach, in which the likelihood is directly approximated, is possible if all populations are small [2] or if the model shows simple dynamics (e.g. multi-dimensional normal distribution with time-dependent mean and covariance matrix) such that the likelihood can be approximated by a normal distribution [3]. In this case, the likelihood (and its derivatives) can usually be approximated efficiently and global optimization techniques are employed to find parameters that maximize the likelihood. However, if large populations are present in the system then direct approximations of the likelihood are unfeasible since the underlying system of differential equations contains one equation for each state and the main part of the probability mass of the model distributes on an intractably large number of states. Similarly, if the system shows complex dynamics such as multimodality, approximations of the likelihood based on Gaussian distributions become inaccurate.

In the last years several methods have been developed to accurately simulate the moments of the underlying probability distribution up to a certain order *k* over time [4–6]. The complexity of these simulation methods is therefore independent of the population sizes but, for large *k*, the corresponding differential equations may become stiff and lead to poor approximations. However, reconstructions of complex distributions from their moments show that for many systems already for small *k* (e.g. *k*∈{4,…,8}) the moments contain sufficient information about the distribution such as the strength and location of regions of attraction (i.e. regions of the state space containing a large proportion of the probability mass) [7].

For models with complex distributions such as multiple modes or oscillations, the accuracy and the running time of the moment approximation can be markedly improved, when conditional moments are considered in combination with the probabilities of appropriately chosen system modes such as the activity state of the genes in a gene regulatory network [8–11]. Recently a full derivation of the conditional moment equations was derived and numerical results show that when the maximum order of the considered moments is high, the number of equations that have to be integrated is usually much smaller for the conditional moments approach and the resulting equations are less stiff [12]. In addition, the approximated (unconditional) moments are more accurate when the same maximal order is considered.

An obvious parameter inference approach is the matching of the observed sample moments with those of the moment-based simulation of the model. Defining the differences between sample and (approximated) population moments as cost functions that depend on the parameters, an approach that minimizes the sum of the squared cost functions seems reasonable. However, in a simple least-squares approach low moments such as means and (co-)variances contribute equally to the sum of squared differences as higher moments, whose absolute magnitudes are much higher (even if they are centralized). Moreover, correlations between the different cost functions may exist and thus necessitate an approach where also products of two different cost functions are considered.

The generalized method of moments (GMM) that is widely used in econometrics provides an estimator that is computed after assigning appropriate weights to the different cost function products [13]. The GMM estimator has, similar as the ML estimator, desirable statistical properties such as being consistent and asymptotically normally distributed. Moreover, for optimally chosen weights it is an asymptotically efficient estimator, which implies that (asymptotically) it has minimum variance among all estimators for the unknown parameters.

In this paper we explore the usefulness of the GMM for moment-based simulations of stochastic reaction networks. We focus on two particular estimators that are commonly used in econometrics: the two-step estimator of Hansen [13] and the demean estimator [14]. We study the accuracy and variance of the estimator for different maximal moment orders and different sample sizes by applying the GMM to two case studies. In addition, we show that poor approximations of some higher order moments have a strong influence on the quality of the estimation. Interestingly, we see that the additional information about the covariances of the cost functions can lead to identification of all parameters. In addition, the variance of the estimator becomes smaller when higher order moments are included. Compared to the simple least-squares approach, the GMM approach yields very accurate estimates.

## Methods

### Stochastic chemical kinetics

Our inference approach relies on a Markov modeling approach that follows Gillespie’s theory of stochastic chemical kinetics. We consider a well-stirred mixture of *n* molecular species in a volume with fixed size and fixed temperature and represent it as a discrete-state Markov process {**X**(*t*),*t*≥0} in continuous-time [15]. The random vector **X**(*t*)=(*X*_1_(*t*),…,*X*_*n*_(*t*)) describes the chemical populations at time *t*, i.e., *X*_*i*_(*t*) is the number of molecules of type *i*∈{1,…,*n*} at time *t*. Thus, the state space of **X** is $\mathbb {Z}^{n}_{+}=\{0,1,\ldots \}^{n}$. The state changes of **X** are triggered by the occurrences of chemical reactions. Each of the *m* different reaction types has an associated non-zero change vector $\mathbf {v}_{j}\in \mathbb {Z}^{n}$ (*j*∈{1,…,*m*}), where $\mathbf {v}_{j}=\mathbf {v}_{j}^{-}+\mathbf {v}_{j}^{+}$ such that $\mathbf {v}_{j}^{-}$ ($\mathbf {v}_{j}^{+}$) contains only non-positive (non-negative) entries and specifies how many molecules of each species are consumed (produced) if an instance of the reaction occurs, respectively. Thus, if **X**(*t*)=**x** for some $\mathbf {x}\in \mathbb {Z}^{n}_{+}$ with $\mathbf {x}+\mathbf {v}_{j}^{-}$ being non-negative, then **X**(*t*+*dt*)=**x**+**v**_*j*_ is the state of the system after the occurrence of the *j*-th reaction within the infinitesimal time interval [*t,t*+*dt*). W.l.o.g. we assume here that all vectors **v**_*j*_ are distinct.

We use *α*_1_,…,*α*_*m*_ to denote the propensity functions of the reactions, where *α*_*j*_(**x**)·*dt* is the probability that, given **X**_*t*_=**x**, one instance of the *j*-th reaction occurs within [*t,t*+*dt*). Assuming law of mass action kinetics, *α*_*j*_(**x**) is chosen proportional to the number of distinct reactant combinations in state **x**. An example is given in Table 1, where the first reaction gives as change vectors, for instance, $\mathbf {v}_{1}^{-} = (-1,0,0), \mathbf {v}_{1}^{+} = (0,1,0), \mathbf {v}_{1}=(-1,1,0)$. Note that, given the initial state **x**=(1,0,0), at any time either the DNA is active or not, i.e. *x*_1_=0 and *x*_2_=1, or *x*_1_=1 and *x*_2_=0. Moreover, the state space of the model is infinite in the third dimension. Although our inference approach can be used for any model parameter in the sequel we simply assume that the proportionality constants *c*_*j*_ are unknown and have to be estimated based on experimental data.

**Table 1:** Simple gene expression model [44]: The evolution of the molecular populations DNA _ON_, DNA _OFF_, and mRNA is described by the random vector **X**(*t*) = (*X*_1_(*t*), *X*_2_(*t*), *X*_3_(*t*)), respectively

Reactions	Propensities	Intervals
DNA _ON_ → DNA _OFF_	*α*_1_(**x**)=*b*·*x*_1_	*b*∈[0,0.5]
DNA _OFF_→ DNA _ON_	*α*_2_(**x**)=*a*·*x*_2_	*a*∈[0,0.5]
DNA _ON_ → DNA _ON_+ mRNA	*α*_3_(**x**)=*c*·*x*_1_	*c*∈[0,0.5]

For $\mathbf {x}\in \mathbb {Z}^{n}_{+}$ and *t*≥0, let *p*_*t*_(**x**) denote the probability *P*(**X**(*t*)=**x**). Assuming fixed initial conditions *p*_0_ the evolution of *p*_*t*_(**x**) is given by the chemical master equation (CME) [1]

$$\begin{array}{rcl} \frac{\partial}{\partial t}p_{t}(\mathbf{x})=\!\!\sum\limits_{j:\mathbf{x}\,-\,\mathbf{v}_{j}^{-}\ge 0}\! \alpha_{j}(\mathbf{x}\,-\,\mathbf{v}_{j}) p_{t}(\mathbf{x}\,-\,\mathbf{v}_{j})-\alpha_{j}(\mathbf{x}) p_{t}(\mathbf{x}), \end{array} $$

which is an ordinary first-order differential equation that has a unique solution under certain mild regularity conditions. Since for realistic systems the number of states is very large or even infinite, applying standard numerical solution techniques to the CME is infeasible. If the populations of all species remain small (at most a few hundreds) then the CME can be efficiently approximated using projection methods [16, 17] or fast uniformization methods [18, 19]. Otherwise, i.e., if the system contains large populations, then analysis methods with running times independent of the population sizes have to be used such as moment closure approaches [4–6] or methods based on van Kampen’s system size expansion [20, 21]. For both approaches, accurate reconstructions of the underlying probability distribution, i.e., the solution of the CME, are possible [7, 21].

### Moment-based analysis

From the CME it is straightforward to derive the following equation for the derivative of the mean of a polynomial function $T: \mathbb {Z}^{n}_{+} \to \mathbb {R}$ on **X**(*t*).

1 $$ \begin{array}{rl} & \frac{d}{dt}E[T{(\mathbf{X}(t))}] \\ =& \sum\limits_{{j}=1}^{m}\! E\left[\alpha_{j}(\mathbf{X}(t))\! \cdot\! \left(T(\mathbf{X}(t) + v_{j}) - T(\mathbf{X}(t)) \right)\right] \end{array}  $$

Omitting the argument *t* of **X** and choosing $T(\mathbf {X})=X_{i}, {X_{i}^{2}},\ldots $ yields the following equations for the (exact) time evolution of the *k*-th moment $E[{X}_{i}^{k}]$ of the distribution for the *i*-th species.

2 $$ \begin{array}{rl} & \frac{d}{dt}E[{({X}_{i})^{k}}] \\ =& \sum\limits_{{j}=1}^{m} E[{\alpha_{j}(\mathbf{X}) \cdot \left(({X}_{i} + v_{ji})^{k} - ({X}_{i})^{k} \right)}], \end{array}  $$

where *v*_*ji*_ refers to the *i*-th component of the change vector **v**_*j*_. In a similar way, equations for mixed moments are derived.

If all reactions are at most monomolecular ($1\ge \sum _{i} |v_{ji}^{-}|$ for all *j*), then no moments of order higher than *k* appear on the right side (also in the mixed case) and we can directly integrate all equations for moments of at most order *k*. However, most systems *do* contain bimolecular reactions (in particular those with complex behavior such as multistability). In this case we consider a Taylor expansion of the multivariate function

$$f(\mathbf{X})={\alpha_{j}(\mathbf{X}) \cdot \left(T(\mathbf{X} + v_{j}) - T(\mathbf{X}) \right)} $$

about the mean *μ*:=*E*[**X**]. It is easy to verify that, when applying the expectation to the Taylor sum, the right side only contains derivatives of *f* at **X**=*μ*, which are multiplied by central moments of increasing order. For instance, for *k*=1 and a single species system with *n*=1, Eq. (2) becomes

$$\begin{array}{lcl} \frac{d}{dt}E[{({X}_{i})}] &=& \sum\limits_{{j}=1}^{m} v_{ji} E[\alpha_{j}(\mathbf{X})]\\ &=& \sum\limits_{{j}=1}^{m} v_{ji} \left(\alpha_{j}(\mu) +\frac{E[(\mathbf{X}-\mu)]}{1!} \cdot \frac{\partial}{\partial x}\alpha_{j}(\mu) \right.\\ && \left.+\frac{E[(\mathbf{X}-\mu)^{2}]}{2!} \cdot \frac{\partial^{2}}{\partial x^{2}}\alpha_{j}(\mu)+\ldots\right) \end{array} $$

In the expansion, central moments of higher order may occur. For instance, in the case of bimolecular reactions, the equations for order *k* moments involve central moments of order *k*+1 since second order derivatives are non-zero. By converting the non-central moments to central ones and truncating the expansion at some fixed maximal order *k*, we can close the system of equations when we assume that higher order central moments are zero. A full derivation of the moment equations using multi-index notation (as required for *n*>1) can be found in [6].

The accuracy of the inference approach that we propose in the sequel depends not only on the information given by the experimental data but also on the accuracy of the approximated moments. Different closure strategies have been suggested and compared in the last years showing that the accuracy can be improved by making assumptions about the underlying distribution (e.g. approximate log-normality) [22, 23]. In addition, the accuracy of moment-closure approximations has been theoretically investigated [24].

### Hybrid approaches

Compared to deterministic models that describe only average behaviors, stochastic models provide interesting additional information about the behavior of a system. Although this comes with additional computational costs, it is in particular for systems with complex behavior, such as multimodality or oscillations, of great importance. Often the underlying source of multiple modes are discrete changes of gene activation states that are described by chemical species whose maximal count is very small (e.g. 1 for the case that the gene is either active, state 1, or inactive, state 0). Then the moment-based approaches described above can be improved (both in terms of accuracy and computation time) by considering conditional moments instead [8–10, 12, 25]. The idea is to split the set of species into species with small and large populations and consider the moments of the large populations conditioned on the current count of the small populations. For the small populations, a small master equation has to be solved additionally to the moment equations to determine the corresponding discrete distribution. More specifically, if $\hat {\mathbf {x}}$ is the subvector of **x** that describes the small populations and $\tilde {\mathbf {x}}$ is the subvector of the large populations (i.e. $\mathbf {x}=(\hat {\mathbf {x}},\tilde {\mathbf {x}})$), then for the distribution of $\hat {\mathbf {x}}$ we have

$$\begin{array}{rcl} \frac{d}{dt} p_{t}(\hat{\mathbf{x}}) &=& \sum\limits_{j:\hat{\mathbf{x}}-\hat{\mathbf{v}}_{j}\ge 0} E[\alpha_{j}(\mathbf{X})\mid \hat{\mathbf{X}}=\hat{\mathbf{x}}-\hat{\mathbf{v}}_{j}] p_{t}(\hat{\mathbf{x}}-\hat{\mathbf{v}}_{j}) \\ && - \sum_{j} E[\alpha_{j}(\mathbf{X})\mid \hat{\mathbf{X}}=\hat{\mathbf{x}}] p_{t}(\hat{\mathbf{x}}) \end{array} $$

where $\hat {\mathbf {v}}_{j}$ is the corresponding subvector of **v**_*j*_. Using Taylor expansion, the conditional expectations of the propensities can, as above, be expressed in terms of conditional moments of the large populations. In addition, equations for the conditional moments of the large populations can be derived in a similar way as above. For instance, the partial mean $E[\tilde X_{i} \mid \hat x] p_{t}(\hat x)$ follows the time evolution

$$\begin{array}{l} \frac{\partial}{\partial t} \left(E[\tilde X_{i} \mid \hat x] p_{t}(\hat x) \right) \\ \,\quad =\!\! \sum\limits_{j:\hat{\mathbf{x}}-\hat{\mathbf{v}}_{j}\ge 0}\!\! E[(\tilde X_{i}+v_{ij})\alpha_{j}(\mathbf{X})\mid \hat{\mathbf{X}}=\hat{\mathbf{x}}-\hat{\mathbf{v}}_{j}] p_{t}(\hat{\mathbf{x}}-\hat{\mathbf{v}}_{j}) \\ \,\quad - \sum_{j} E[\tilde X_{i}\alpha_{j}(\mathbf{X})\mid \hat{\mathbf{X}}=\hat{\mathbf{x}}] p_{t}(\hat{\mathbf{x}}) \end{array} $$

where on the right side again Taylor expansion can be used to replace unknown conditional expectations by conditional moments. As above a dependence on higher conditional moments may arise and a closure approach has to be applied to arrive at a finite system of equations. Unconditional moments can then be derived by summing up the weighted conditional moments. It is important to note that if $p_{t}(\hat {x})=0$ then algebraic equations arise turning the equation system into a system of differential-algebraic equations, which renders its solution more difficult (see [12, 26] for details).

**Fig. 1:**
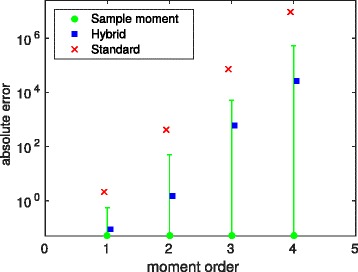
Absolute error of the first four moments of P_1_ for the exclusive switch model, where the moments are either computed based on a standard moment closure approach or a hybrid approach. The maximal order of the considered moments is 5

In Fig. 1 we give an example for a comparison of the accuracy of the hybrid approach and the standard moment closure (assuming that all central moments above a fixed maximal order are zero) for one of our case studies. As “exact” moment values we chose the average of 500,000 samples generated by the stochastic simulation algorithm (SSA) [27] and considered the absolute difference to the approximated moments of one chemical population until a maximal order of four. Since for our case studies we assumed 10,000 samples we additionally plot the (approximated) standard deviation of the 50 sample means taken from batches of 10,000 samples. The moments computed based on the hybrid approach show a smaller error than those computed using the standard moment closure and lie within the deviations given by the sample moments. For the example in Fig. 1 we have 126 equations for the standard approach up to an order of four. In the hybrid case there are 14 moment equations and one equation for the mode probability per mode leading to a total number of 45 equations. However, reductions are possible for the standard approach when the model structure is exploited [28]. We do not make use of these reductions here but choose the hybrid approach mainly because it gives more accurate results for the (unconditional) moments. This strongly improves the quality of the estimated parameters as demonstrated in the “Results” section.

### Generalized method of moments

We assume that observations of a biochemical network were made using single-cell analysis that gives population snapshot data (e.g. from flow cytometry measurements). Typically, large numbers (about 5,000–10,000 [29–31]) of independent samples can be obtained where each sample corresponds to one cell. It is possible to simultaneously observe one or several chemical populations at a time in each single cell. In the sequel, we first describe the inference procedure for a single observation time point and a single chemical species that is observed. Later, we extend this to several time points and species.

For a fixed measurement time *t* and a fixed index *i* of the observed population we can define the *r*-th order sample moment as

$$\hat m_{r} = \frac{1}{N} \sum_{\ell=1}^{N} Y_{\ell}^{r}, $$

where *Y*_*ℓ*_ is the *ℓ*-th sample of the observed molecular count of the *i*-th species at time *t* and there are *N* samples in total. For large *N*, the sample moments are asymptotically unbiased estimators of the population moments.

Let *θ* be a vector of, say, *q*≤*m* unknown reaction rate constants^1^, for which some biologically relevant range is known. Moreover, let *m*_*r*_ be the *r*-th theoretical moment, i.e., $m_{r}(\theta):=E[Y_{\ell }^{r}]$. In the sequel we also simply write *Y* instead of *Y*_*ℓ*_ whenever *Y* appears inside the expectation operator or when the specific index of the sample is not relevant. An obvious inference approach would be to consider the ordinary least squares estimator

3 $$\begin{array}{@{}rcl@{}} \hat{\theta} &=&\arg\min_{\theta} \sum\limits_{r=1}^{k} \left(\hat{m}_{r}-m_{r}(\theta)\right)^{2}, \end{array} $$

where *k* is the number of moment constraints. Under certain conditions related to the identification of the parameters as discussed below, this estimator is consistent (converges in probability to the true value of *θ*) and asymptotically normal. However, its variance may be very high. This is due to the fact that for increasing order the variance of the sample moments increases and so does the variance of the estimator. This problem can be mitigated by choosing appropriate weights for the summands in (3). Moreover, since correlations between the cost functions

$$g_{r}(\theta)=\hat m_{r}-m_{r}(\theta) $$

exist, a more general approach that considers mixed terms is needed. This leads to a class of estimators, called generalized method of moments (GMM) estimators that have been introduced by Hansen [13]. The idea is to define the estimator as

4 $$\begin{array}{@{}rcl@{}} \hat{\theta} &=&\arg\min_{\theta} \mathbf{g}(\theta)' W \mathbf{g}(\theta) \end{array} $$

where **g**(*θ*) is the column vector with entries *g*_*r*_(*θ*),*r*=1,…,*k*, and *W* is a positive semi-definite weighting matrix. Note that by defining *f*_*r*_(*Y*,*θ*)=*Y* ^*r*^−*m*_*r*_(*θ*) we see that

$$g_{r}(\theta)=\frac{1}{N}\sum_{\ell} f_{r}(Y_{\ell},\theta)=\frac{1}{N}\sum_{\ell} Y_{\ell}^{r} -m_{r}(\theta) $$

is the sample counterpart of the expectation *E*[*f*_*r*_(*Y*,*θ*)]. The latter satisfies

$$\theta_{0}= \arg\min_{\theta} E[\mathbf{f}(Y,\theta)]' W E[\mathbf{f}(Y,\theta)], $$

where **f**(*Y*,*θ*) is the column vector with entries *f*_*r*_(*Y*,*θ*) and *θ*_0_ is the true value of *θ*. Note that the choice *W*=*I* gives the least-squares estimator with *k* terms while for general *W* there are $\frac {k\cdot (k+1)}{2}$ terms in the objective function (with *k* being the dimension of **g**(*θ*)). In addition, we remark that in general *W* may depend on *θ* and/or the samples *Y*_*ℓ*_.

Here we assume that identification of *θ* is possible, i.e., we require that *q*≤*k*, i.e., the number of the moment constraints used is at least as large as the number of unknown parameters and

$$E[\mathbf{f}(Y,\theta)]=\mathbf{0}~\text{if and only if}~\theta=\theta_{0}. $$

In addition, the theoretical moments *m*_*r*_(*θ*) should not be functionally dependent (see Chapter 3.3 in [32]) to ensure that the information contained in the moment conditions is sufficient for successfully identifying the parameters.

By applying the central limit theorem to the sample moments, it is possible to show that the GMM estimator is consistent and asymptotically normally distributed and that its variance becomes asymptotically minimal if the matrix *W* is chosen such that it is proportional to the inverse of the covariances between the $Y_{\ell }^{r}$ [13]. This result is intuitive since usually higher moments might be more volatile than others and, thus, it makes sense to normalize the errors in the moments by the corresponding covariance. Formally, we define **Y**_*ℓ*_ as the random vector with entries (*Y*_*ℓ*_)^*r*^ for *r*=1,…,*k* and, as before, omit the subindex *ℓ* if it is not relevant. Then,

$$F(\theta_{0})=\mathit{COV}[\mathbf{Y},\mathbf{Y}]=E[\mathbf{f}({Y},\theta_{0})\mathbf{f}({Y},\theta_{0})^{T}] $$

and choosing *W*∝*F* ^−1^ will give an estimator with smallest possible variance, i.e., it is asymptotically efficient in this class of estimators [13, 32].

Since *F* depends on the true value *θ*_0_, a two-step updating procedure has been suggested [13] during which *W* is chosen as the identity matrix *I* in the first step such that an initial estimate $\tilde \theta $ is computed. In a second step, *F* is estimated by the sample counterpart of $E[\mathbf {f}({Y},\tilde \theta)\mathbf {f}({Y},\tilde \theta)^{T}],$ i.e.,

5 $$ \hat{F}_{1}(\tilde\theta)=\frac{1}{N}\sum^{N}_{\ell=1}\mathbf{f}({Y}_{\ell},\tilde\theta)\mathbf{f}({Y}_{\ell},\tilde\theta)^{T}.  $$

If, however, the model is “misspecified”, i.e., there is no *θ*_0_ for which

$$E[\mathbf{f}({Y},\theta_{0})] = \mathbf{0}, $$

then the above estimator is no longer consistent. In particular, if the theoretical moments are poorly approximated, it is likely that also the accuracy of the resulting estimates is poor. An estimator for *F* that is consistent is then given by [32]

6 $$ \hat{F}_{2}=\frac{1}{N}\sum^{N}_{\ell=1} (\mathbf{Y}_{\ell} - \overline{\mathbf{Y}})(\mathbf{Y}_{\ell} - \overline{\mathbf{Y}})^{T},  $$

where $ \overline {\mathbf {Y}}$ is the vector with entries $\frac {1}{N}\sum _{\ell =1}^{N} \mathbf {Y}_{\ell }^{r}$. In the sequel we refer to the estimator based on (6) as the *demean estimator*. This estimator removes the inconsistencies in the covariance matrices estimated from the sample moments by “demeaning”. Since moment-based analysis methods usually give approximations of the moments and not the exact values, we consider both, the demean estimator defined by (6) and the estimator of the 2-step procedure in (5) for our numerical results.

The estimation procedure described above can be generalized to several dimensions by also using mixed sample moments instead of only $\hat {m}_{r} $ and mixed theoretical moments instead of only *m*_*r*_(*θ*). For instance, for moments up to order two and two simultaneously observed species *X* and *Y*, we use the cost functions

$$\begin{array}{rcl} g_{1}(\theta)&=&\frac{1}{N}\sum_{\ell=1}^{N} X_{\ell} - E[X_{\ell}\mid\theta] \\ g_{2}(\theta)&=&\frac{1}{N}\sum_{\ell=1}^{N} Y_{\ell} - E[Y_{\ell}\mid\theta] \\ g_{3}(\theta)&=&\frac{1}{N}\sum_{\ell=1}^{N} X_{\ell} Y_{\ell} - E[X_{\ell} Y_{\ell}\mid\theta] \\ g_{4}(\theta)&=&\frac{1}{N}\sum_{\ell=1}^{N} X_{\ell}^{2} - E[X_{\ell}^{2}\mid\theta] \\ g_{5}(\theta)&=&\frac{1}{N}\sum_{\ell=1}^{N} Y_{\ell}^{2} - E[Y_{\ell}^{2}\mid\theta]. \\ \end{array} $$

In the same way, we can extend the estimators $\hat {F}_{1}$ and $\hat {F}_{2}$ to several dimensions. For instance, the covariance between *X*_*ℓ*_ *Y*_*ℓ*_ and $X_{\ell }^{2}$ can be estimated as

$$\frac{1}{N}\sum_{\ell=1}^{N} (X_{\ell} Y_{\ell} - \overline{XY})(X_{\ell}^{2} - \overline{X^{2}}), $$

where again we use $\overline {*}$ to denote the sample mean operator.

If, instead of snapshot data for a single observation time, independent samples for different times are available then the GMM estimator can also be easily generalized to

7 $$\begin{array}{@{}rcl@{}} \hat{\theta} &=&\arg\min_{\theta} \sum_{t=t_{0}}^{t_{f}} \mathbf{g}^{(t)}(\theta)' W^{(t)} \mathbf{g}^{(t)}(\theta). \end{array} $$

Here, for each time point *t*∈{*t*_0_,…,*t*_*f*_} the vector of cost functions **g** ^(*t*)^ is calculated as before and the minimum is taken over the sum of these uncorrelated cost functions. Note that for each observation time point a weight matrix *W* ^(*t*)^ has to be computed. In the two-step approach, the initial weight matrices are all equal to the identity matrix and then in the second step different weight matrices may arise since the estimator of *F* depends on *Y*, which in turn depends on the distribution of the model at the specific time *t*.

## Results

To analyze the performance of the GMM we consider two case studies (see Additional file 1), the simple gene expression model in Table 1 and a network of two genes with mutual repression, called exclusive switch [33]. The reactions of the exclusive switch are listed in Table 2. All propensities follow the law of mass action. For the parameters that we chose, the corresponding probability distribution is bi-modal.

**Table 2:** Exclusive switch model [33]: Two different proteins P_1_ and P_2_ can bind to a promotor region on the DNA. If P_1_ is bound to the promotor the production of P_2_ is inhibited and vice versa. In the free state both proteins can be produced

Reactions, *i*=1,2			Rate constant	Interval
DNA	→	DNA + P _*i*_	*p*_*i*_	[0.5,1.5]
DNA.P _*i*_	→	DNA.P _*i*_ + P _*i*_	*p*_*i*_	[0.5,1.5]
P _*i*_	→	*∅*	*d*_*i*_	[0,0.05]
DNA + P _*i*_	→	DNA.P _*i*_	*b*_*i*_	[0,0.1]
DNA.P _*i*_	→	DNA + P _*i*_	*u*_*i*_	[0,0.1]

For fixed reaction rate constants and initial conditions, we used the SSA to generate trajectories of the systems and record samples of the size of the corresponding protein/mRNA populations. In addition, we used the software tool SHAVE [34] to generate moment equations both for the standard moment closure and for the hybrid approach. In SHAVE the partial moments are integrated instead of the conditional moments such that the differential-algebraic equations are transformed into a system of (ordinary) differential equations after truncating modes with insignificant probabilities. Then an accurate approximation of the solution using standard numerical integration methods can be obtained. The system of moment equations is always closed by setting all central moments of order >*k* to zero. We used for the inference approach only the moments up to order *k*−1 since the precision of the moments of highest order *k* is often poor. SHAVE allows to export the (hybrid) moment equations as a MATLAB-compatible m-file. We then used MATLAB’s ode45 solver, which is based on a fifth order Runge-Kutta method, to integrate the (hybrid) moment equations. Note that for the gene expression example, the moment equations are exact since all propensities are linear. Thus, even an analytic solution is possible for this system.

We then used MATLAB’s Global Search routine to minimize the objective function in Eq. (4). Global Search is a method for finding the global minimum by starting a local solver from multiple starting points that are chosen according to a heuristic [35]. Therefore the total running time of our method depends on the tightness of the intervals that we use as constraints for the unknown parameters as well as on the starting points of the Global Search procedure. The running times for one local solver call (using the hybrid approach for computing moments) were about 2 s (demean estimator) and 40 s (2-Step estimator) for the gene expression model. For the exclusive switch the average running time for a local solver call was about 2 min (demean) and 10 min (2-Step). Note that the total running time depends on the amount of local solver calls carried out by Global Search, which varied between 2 and 50. For all experiments we chose a single initial point that is located far away from the true values and allowed Global Search to choose 500 (potential) further starting points. Different initial points yielded similar results except if the initial points is chosen close to the true values (then the results are significantly better in particular in the case of only few moment constraints).

The intervals that we used as constraints for the parameters are all listed in Tables 1 and 2.

### Standard vs. hybrid moment-based analysis

**Fig. 2:**
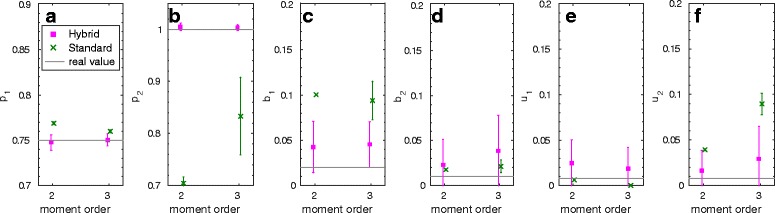
Exclusive switch model: Comparison of estimations for 6 parameters (**a**–**f**) with the demean procedure for the standard moment closure and hybrid moments

In Fig. 2 we plot the results of a comparison between the standard and the hybrid moment closure when it is performed during the optimization procedure of the GMM inference approach. We chose the exclusive switch model for this since for this model the accuracy of the standard approach is poor. As an estimator for *F* we used (6), which is based on demeaning (demean). Results for the 2-step procedure show similar differences when standard and hybrid moment closure are compared. We fixed the degradation rates to ensure that identification of *p*_1_ and *p*_2_ is possible when the two protein populations are measured at only a single observation time point. To simultaneously identify all parameters (including *p*_1_ and *p*_2_) several observation time points are necessary (see Additional file 2).

The true values of the six unknown parameters are plotted against the means and standard deviations of the estimated values for a maximal moment order of 2 and 3, where for each of the six unknown parameters 50 estimations based on 10,000 samples each were used.

We see that the inaccurately approximated moments of the standard approach lead to severe problems in the inference approach. Nearly all parameters are estimated more accurately when the hybrid moment closure is used. For parameter *b*_1_ most of the optimization runs converged to the upper limit of the given interval (0.1) when the standard approach was used. For the results in the sequel, we only used the hybrid moment closure.

### Two-step vs. demean approach

**Fig. 3:**
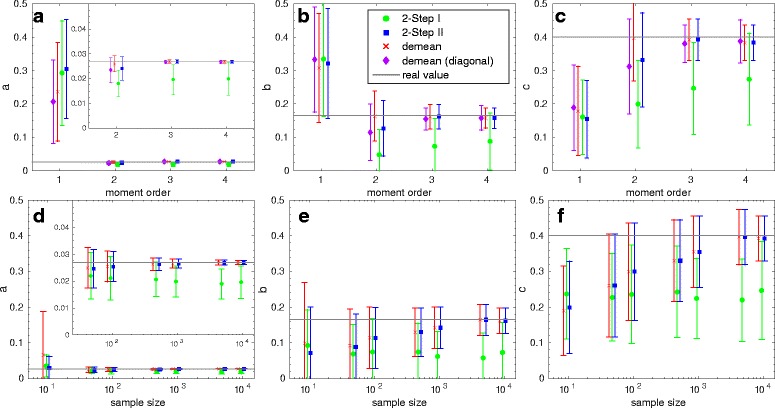
Gene expression model: Estimated parameters *a,b* and *c* for different numbers/orders of moments and 10,000 samples (**a**-**c**) and for different sample sizes based on 3 moments (**d**-**f**). The inner plots show results on a more detailed scale (**a** and **d**)

**Fig. 4:**
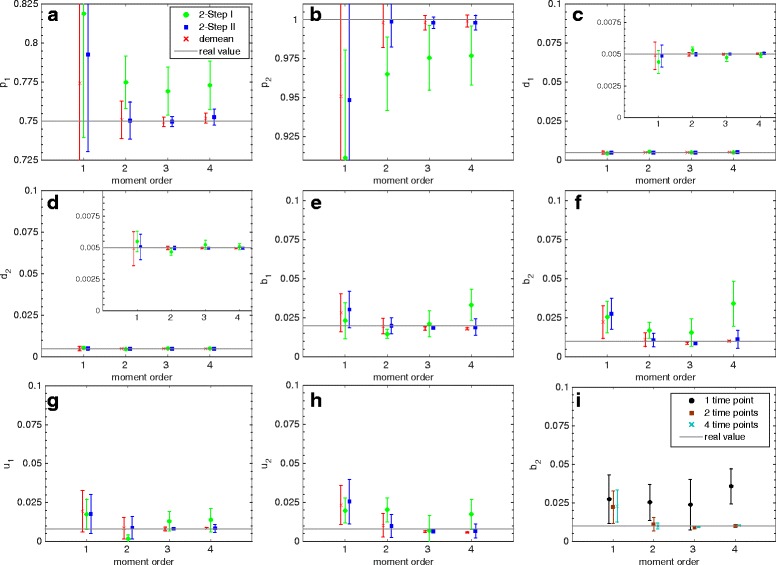
Exclusive switch model: Estimated parameters for maximal moment order 1–4 based on 10,000 independent samples observed at time *t*=100 and *t*=200 (**a**–**h**) and at 1–4 different time points for the demean-based estimation of *b*_2_ (**i**). The inner plots show results on a more detailed scale (**c** and **d**)

In Figs. 3 and 4 we plot results of the GMM approach applied to the two example networks, where we compare the performance of the two-step estimator in Eq. (5) with the demean estimator in Eq. (6). We plot the true values of the parameters against the estimated values, where 2-Step I is the result of the first step of the two-step procedure (with *W*=*I*) and 2-Step II that of the second step (with $W=\hat F_{1}$ and $\hat F_{1}$ as defined in Eq. (5)).

For the results in Fig. 3 only one population (mRNA) was observed at *t*=100 where the initial conditions were such that DNA _OFF_=1, DNA _ON_=0 and 10 mRNA molecules were present in the system. For three parameters the means and standard deviations of the estimated values are plotted, again based on 50 repetitions of the inference procedure.

In the first row of Fig. 3 the accuracy of the estimation is compared with respect to the number/order of moments considered, where again for each of the 50 estimated values 10,000 samples were used. We see that if only one moment is considered or if equal weights are used for the first two moments, only a rough estimate is possible since identification is not possible. The accuracy is markedly improved when the weights are chosen according to the demean approach. Here, it is important to note that for a maximal order of *k*=2, in *W* we also consider, besides the squared cost functions *g*_1_(*θ*)^2^ and *g*_2_(*θ*)^2^, the mixed term *g*_1_(*θ*)*g*_2_(*θ*). This additional term significantly improves the quality of the estimation such that it is possible to achieve a good estimation of the parameters with only the sample mean and the sample second moment. To further investigate the positive influence of the mixed term, we additionally plot results for the case that only variances are estimated, referred to as ‘demean (diagonal)’, i.e., the weight matrix is the inverse of a diagonal matrix that contains the variances estimated based on the demean approach.

However, the variance of the estimator for a maximum order of two is relatively high but decreases significantly when also the third (and fourth) moment is considered. Here, demean and the second step of the two-step procedure perform equally well and also demean (diagonal) gives very good results. Opposed to this *W*=*I* (first step of two-step procedure) gives poor results and a high variance also if higher moments are considered.

In Table 3 we give an example for the (normalized) matrix *W* as used for demean and 2-Step II. The two methods choose nearly identical weights and the mean has the highest weight. Then, the mixed cost function for mean and second moment has a (negative) weight of about 2·(−4.95) % since these moments are negatively correlated (and so are the second and third moment). All terms that involve the third moment have a very small weight as their covariances are high.

**Table 3:** Weight matrices for the two-step and demean procedure with moment order 3 for the gene expression model

*W*			
Two-step	1	-0.0495	0.0007
	–0.0495	0.0025	–3.86 *e* ^−5^
	0.0007	–3.86 *e* ^−5^	6.11 *e* ^−7^
Demean	1	–0.0494	0.0007
	–0.0494	0.0025	–3.85 *e* ^−5^
	0.0007	–3.85 *e* ^−5^	6.09 *e* ^−7^

The entries are normalized with respect to the weight for the mean and rounded (the original weight matrices are both positive semi-definite)

It is important to note that also if the number of moment constraints, *k*, is equal to the number of parameters, *q*, 2-Step I performs poor (see results for maximal order *k*=3 in the first row of Fig. 3). The reason is that in this example identification is not possible if only three terms are used due to functional dependencies between the parameters of the first two reactions and due to the fact that only at a single time point measurements were made. If identification was possible and the computed population moments were exact, the results should be independent of the choice of *W* for the case that *q* equals *k*.

Thus, the weights given by the estimators for *F* in (5) and (6) substantially increase the accuracy of the results and allow identification, because additional information about the covariances between the *Y* ^*r*^ are used. Moreover, due to the off-diagonal entries of *W* additional mixed terms are part of the objective function.

In the second row in Fig. 3, we compare the accuracy for different samples sizes where the first three moments were considered. While 2-Step I does not show a systematic improvement when the number of samples increases, we see for 2-Step II and demean not only significantly improved estimates but also smaller variances. However, in the case of few samples, demean gives in particular for parameter *a* a high variance. This comes from the fact that the corresponding estimator uses the sample mean instead of the theoretical mean and therefore the weight matrix is far from optimal if *N* is small.

In Fig. 4, **a**–**h**, we plot results for the exclusive switch model where all eight parameters were estimated based on observations of the two protein populations of *P*_1_ and *P*_2_ at two time points. On the x-axis the maximal order of moments used is plotted. For the orders 1, 2, 3 and 4 there are in total 2, 5, 9 or 14 moments, respectively. Again, 2-Step II and demean both give accurate results from a maximal order of two on, whereas 2-Step I gives poor results. In addition, the variance of the estimator decreases with increasing maximal order. However, the values for 2-Step II become slightly worse and have higher variance for a maximal order of four since these moments are not approximated very accurately. Also the accuracy of the demean estimator does not improve when the maximum order is increased from three to four. Thus, the cost functions of order four moments do not lead to any significant improvement in this example and should be excluded.

### Further estimators

For our results we focused on the most popular GMM estimators, that is, demean and two-step. However, we also implemented two additional variants of estimators that are frequently described in the GMM literature [14, 36]. One is the estimator that results from further iterations of the two-step procedure (iterated GMM estimator [36]). However, in our examples we did not see an increase in accuracy after the second iteration. The second approach is the continuously updating GMM estimator [36], where we use in Eq. (4) the weight matrix $W(\theta)=(\hat {F}_{1}(\theta))^{-1}$ of Eq. (5) and the argument *θ* is not fixed for the optimization but optimized simultaneously with the argument of **g**(*θ*). The results for this approach did not show increased accuracy, also when we used results of the other estimators (e.g. demean) as starting points for the optimization. Moreover, for large weight matrices, the recomputation in each step of the optimization resulted in longer running times.

Overall, our experiments show that for sufficiently large *N* the demean estimator usually yields the best results, while two-step performs better for small *N*. Moreover, choosing three as the maximum order gave the best results (accurate average value and small standard deviations) for the examples that we considered.

## Discussions

In the context of stochastic chemical kinetics, parameter inference methods are either based on Markov chain Monte Carlo schemes [37–40], on approximate Bayesian computation techniques [41–43] or on maximum likelihood estimation using a direct approximation of the likelihood [2, 44] or a simulation-based estimate [45, 46]. Maximum likelihood estimators are, in a sense, the most informative estimates of unknown parameters [47] and have desirable mathematical properties such as unbiasedness, efficiency, and normality. On the other hand, the computational complexity of maximum likelihood estimation is high as it requires a simulation-based or numerical solution of the CME for many different parameter instances. Since the applicability of these methods is limited, approaches based on moment closure [3, 23, 48–51] or linear noise approximations [52–54] have been developed. An approximation of the likelihood of order-two sample moments is maximized in [23, 48, 49, 51]. The approach exploits that for large numbers of samples these sample moments are asymptotically normally distributed. The negative log-likelihood leads to an optimization problem where the differences between the sample and theoretical moments up to order two are weighted and minimized as well. As opposed to the GMM, the weight matrix in [48, 49] is estimated based on the theoretical moments of the model up to order four and independent of the samples while in the GMM approach this matrix depends on the samples (and theoretical moments up to order two). Moreover, the objective function contains an additional summand, which is the logarithm of the determinant of the estimated covariance matrix. In [23], Bogomolov et al. insert sample instead of theoretical moments in the derived formulas for the covariances of moment conditions up to order two. A comparison for the two examples that we consider in the previous section yields that when the theoretical moments are used to estimate covariances, similar to the continuously updating GMM, optimization was slow and sometimes failed to return the global optimum due to a much more complex landscape of the objective function. When sample moments are considered as suggested in [23], the results are similar to those of the GMM demean estimator for a maximum order of two. In [51], only variances are considered (weight matrix is diagonal) and estimated based on the samples. Therefore, it does not exploit the information contained in the mixed terms, which lead to improved estimates in our examples (see results for ‘demean (diagonal)’ in Fig. 3).

A similar approach is used in [3] where the moment equations are closed by a Gaussian approximation. The parameter estimation is based on using a ML estimator and a Markov chain Monte-Carlo approach. In [50] the importance of higher moment orders when using least square estimators is shown. Weights for terms that correspond to different moments are chosen ad hoc and not based on any statistical framework.

Here, we present results for the general method of moments that assigns optimal weights to the different moment conditions for an arbitrary maximal moment order and number of species. We showed that trivial weights (e.g. identity matrix) give results whose accuracy can be strongly increased when optimal weights are chosen. In the very common case that functional dependencies between parameters exist (e.g. degradation and production of the same species) and identification is difficult, the GMM estimator allows to accurately identify the parameters. Moreover, our results indicate that the accuracy of the estimation increases when moments of order higher than two are included. A general strategy could be to start with *k*=*q* cost functions (equal to the number of unknown parameters) and increase the maximal order until tests for over-identifying restrictions (e.g. the Hansen test [13]) suggest that higher orders do not lead to an improvement. In this way, cost functions that do not improve the quality of the estimation, such as the fourth order cost functions for the results in Fig. 4, can be identified.

We also found that an accurate approximation of the moments is crucial for the performance of the GMM estimator. Thus, hybrid approaches such as the method of conditional moments [12] or sophisticated closure schemes (e.g. [23]) should be preferred. If all propensities in the network are linear, the moment equations are exact and model misspecification is not an issue. However, for most networks the moments can only be approximated, since the propensities are nonlinear, and hence the model is potentially misspecified. Again, statistical tests can be used to detect model misspecification [32] and equations for higher order moments may be added to the (conditional) moment equations to improve the approximation of the lower order moments.

Finally, we note that the GMM framework can also be applied when the observed molecular counts are subject to measurement errors. It is straight forward to extend the GMM framework to the case of samples *Y*_*ℓ*_+*ε* where the error term *ε* is independent and normally distributed with mean zero.

## Conclusion

Parameter inference for stochastic models of cellular processes demands huge computational resources. The proposed approach based on the generalized method of moments is based on an adjustment of the statistical moments of the model and therefore does not require the computation of likelihoods. This makes the approach appealing for complex networks where stochastic effects play an important role, since the integration of the moment equations is typically fast compared to other computations such as the computation of likelihoods. The method does not make any assumptions about the distribution of the process (e.g. Gaussian) and complements the existing moment-based analysis approaches in a natural way.

Here, we used a multistart gradient-based minimization scheme, but the approach can be combined with any global optimization method. We found that the weights of the cost functions computed by the GMM estimator yield clearly more accurate results than trivial (identical) weights. In particular, the variance of the estimator decreases when moments of higher order are considered. We focused on the estimation of reaction rate constants and, as future work, we plan to investigate how well Hill coefficients and initial conditions are estimated.

An important advantage of the proposed method is that in the economics literature the properties of GMM estimators have been investigated in detail over decades and several variants and related statistical tests are available. We will also check how accurate approximations for the variance of the GMM estimator are [32]. Since we found that when moments of order higher than three are included, the results become slightly worse, we will in addition explore the usefulness of statistical tests for over-identifying moment conditions. In this way, we can ensure that only moments conditions are included that improve the estimation.

## Endnote

^1^ It is straightforward to adapt the approach that we present in the sequel to the case that other unknown continuous parameters have to be estimated.

## Additional files

Additional file 1 Supplementary document. This additional document shows the influence of multiple time points in case of identifying problems. (PDF 126 kb)

Additional file 2 Matlab code and data. This file contains the Matlab codes and samples which were used to produce the results. (GZ 9349 kb)

## Abbreviations

CME: Chemical master equation
GMM: Generalized method of moments
ML: Maximum likelihood
SSA: Stochastic simulation algorithm
