# Supplementary material for: Generalized method of moments for estimating parameters of stochastic reaction networks
Source: BMC Syst Biol. 2016 Oct 21;10:98. doi: 10.1186/s12918-016-0342-8 (PMC5073941; doi:10.1186/s12918-016-0342-8)
Supplement: Additional file 1 — Supplementary document. This additional document shows the influence of multiple time points in case of identifying problems. (PDF 126 kb) [file 12918_2016_342_MOESM1_ESM.pdf]

METHODOLOGY ARTICLE

Supplementary Material to: Generalized Method of Moments for Estimating Parameters of Stochastic Reaction Networks

Alexander Lück and Verena Wolf*

Supplementary Note – Influence of multiple Time Points

For certain pairs of chemical reactions the identifying condition

$$E[\mathbf{f}(Y,\theta)] = \mathbf{0} \text{ if and only if } \theta = \theta_0.$$

is violated when only regarding snapshot data from a single time point. For example in the very simple reaction system

$$\begin{aligned} \emptyset \to A \quad & \text{rate } \lambda, \\ A \to \emptyset \quad & \text{rate } \mu \end{aligned}$$

every combination of λ and μ with $\frac{\lambda}{\mu} = const$ would lead to the same snapshot data for species A at a certain time point.

In order to resolve this problem more information, i.e. snapshot data at several time points (of independent samples to avoid correlation), is needed or one of the parameters has to be fixed. In section "Standard vs hybrid moment-based analysis" in the main article this problem already occured for the exclusive switch: The corresponding rates are production p_i and degradation d_i as well as binding b_i and unbinding u_i. By fixing the degredation rates d_i the estimation of the production rates becomes quite well, whereas b_i und u_i can not be estimated due to the identifying problem.

For the following estimations the demean procedure was used. The 2-Step method showed a similar behavior. With no fixed parameters and only a single time point $t = 200$ nothing can be reliably estimated as indicated in Fig. 1. The estimated values are often far away from the real ones and the variance is also quite high in all cases.

The consideration of a second time point ($t = 100, 200$) resolves the issue in case of sufficient moment conditions, i.e. order 2 or higher. Four time points ($t = 50, 100, 150, 200$) do not further improve the estimation but due to the higher total number of samples (500,000 per time point) the variance is decreased.

*Correspondence: verena.wolf@uni-saarland.de
Department of Computer Science, Saarland University, Campus E 13, 66123 Saarbrücken, Germany
Full list of author information is available at the end of the article

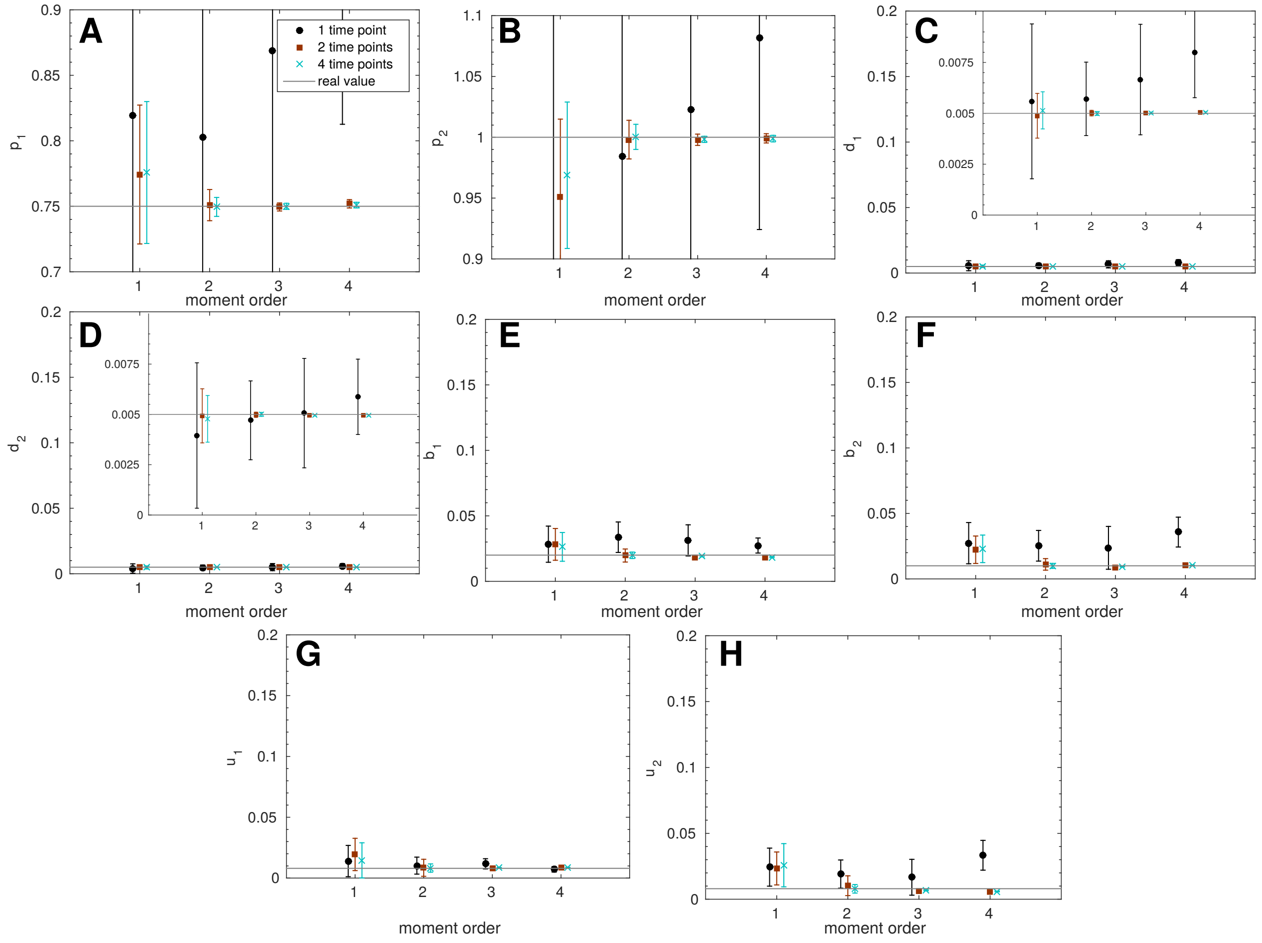

Figure 1 Exclusive switch model: Comparison of estimations with the demean procedure for single time point data and combined data for samples of two and four independent time points.
